# Preclinical In Vitro Evaluation of an Innovative Nutraceutical Mixture Modulating the Histamine H1 Receptor—Preliminary Data

**DOI:** 10.3390/cimb48090854

**Published:** 2026-08-22

**Authors:** Franco Frati, Carlo Cavaliere, Francesco Frati, Giulio Torello, Marianna Colasante, Chiara Caputo, Marta Scquizzato, Simonetta Masieri

**Affiliations:** 1 Private Independent Researcher, 20123 Milano, Italy; francofrati57@gmail.com; 2 Department of Sense Organs, Sapienza University, 00161 Rome, Italy; carlo.cavaliere@uniroma1.it; 3 Clinic of Otolaryngology (ENT), University of Perugia, 06123 Perugia, Italy; francescofrati92@hotmail.it; 4 TL Pharma Consulting, 65129 Pescara, Italy; giulio.torello@tlpharmaconsulting.it (G.T.); ricerca@tlpharmaconsulting.it (M.C.); 5 Science Department Global Pharmacies Partner, 20123 Milano, Italy; c.caputo@gppitaly.it; 6 Department of Oral and Maxillofacial Sciences, Sapienza University, 00161 Rome, Italy; studiomasieri@gmail.com

**Keywords:** allergy, H1 receptor, histamine, drugs, Nutraceuticals

## Abstract

The primary pathogenic mechanism of Type I hypersensitivity reactions involves histamine-mediated activation of the H1 receptor (H1R) and the subsequent signaling of the phospholipase C β-inositol trisphosphate (PLCβ-IP_3_) pathway. The present study evaluates the in vitro efficacy of a novel nutraceutical mixture—Quercetin, *Perilla frutescens*, *Boswellia serrata*, Blackcurrant, *Parthenium*, *Helichrysum*, *Lactobacillus Acidophilus* (*L. acidophilus*) and *Bifidobacterium animalis* (*B. animalis*)—in modulating *HRH1* gene expression and the activation of the intracellular second messenger inositol trisphosphate (IP_3_). Quantitative PCR (qPCR) analysis revealed that the mixture downregulated *HRH1* expression by approximately 33% (*p* < 0.05) compared to the control group. Furthermore, ELISA assays demonstrated that the mixture significantly reduced IP_3_ release by 10.30% (*p* < 0.05 vs. positive control). These results indicate that the nutraceutical mixture exerts its effects by modulating the H1R-PLCβ-IP_3_ pathway through the reduction in both H1 receptor gene expression and IP_3_ release. These findings suggest a potential mechanism of action involving the modulation of the histamine H1 receptor. Consequently, the mixture emerges as a promising candidate for further investigation into the H1R-IP_3_ activation cascade.

## 1. Introduction

Allergy is an unexpected, abnormal or exaggerated reaction to an exogenous stimulus involving the immune system [1]. In the clinical literature, it is crucial to properly position allergy within the broader framework of hypersensitivity, as these terms are not strictly synonymous. Hypersensitivity encompasses objective, reproducible symptoms initiated by exposure to a defined stimulus at a dose tolerated by normal subjects. Allergy, on the other hand, is specifically defined as a subcategory of hypersensitivity reactions that are explicitly mediated by confirmed or highly probable immunological mechanisms. Consequently, non-allergic hypersensitivity represents a broader clinical classification that does not meet the specific immunological criteria required for a true allergy [2]. The prevalence and complexity of allergic diseases and asthma are increasing worldwide, particularly in low- and middle-income countries [3]. The prevalence of physician-diagnosed allergic diseases has increased significantly over time and currently affects approximately 10% to 30% of the global population [4].

Allergic diseases such as allergic rhinitis (AR), allergic asthma (AAS), atopic dermatitis (AD), food allergy (FA), and eczema are systemic diseases caused by an impaired immune system [5].

Allergic rhinitis is no longer a localized concern but a global health problem, increasingly classified by its persistence and severity rather than seasonality, and fundamentally linked to asthma under the unified airway disease concept [6].

Allergic rhinitis is clinically characterized by nasal symptoms, and recent findings suggest that the most significant decline in general health-related quality of life (Qol) is driven by extranasal manifestations: poor sleep quality and otologic symptoms. This decline in QoL exerts a substantially greater impact on the patient’s physical and psychological well-being [7].

In this pathological context, mast cells play a significant role. Within human anatomy, mast cells are distributed throughout the loose connective tissues of every organ, with a particular concentration near nerve fibers and the vascular and lymphatic systems.

Their density is especially high in areas prone to allergic manifestations; notably, within the respiratory system, the integumentary system, the gastrointestinal tract, and reproductive mucosal surfaces [8].

Immunoglobulin E (IgE) and eosinophils play a critical role as key mediators of allergic inflammation that distinguish it from other forms of inflammatory disease. While IgE binds to mast cells and triggers degranulation, eosinophils produce a variety of pro-inflammatory mediators capable of inducing bronchial hyperreactivity.

IgE influences the allergic inflammatory response by interacting with the high-affinity IgE receptor (FcεRI) on mast cells and basophils. The cross-linking of bound IgE to FcεRI by allergen triggers mast cell degranulation, which results in the release of preformed mast-cell inflammatory mediators such as histamine, tryptase, leukotriene and prostaglandin [9]. There are also other mediators released during degranulation, these being the cytokines. Mast cells contain preformed stores of TNFα available for immediate release that might influence IgE-associated allergic inflammation [10].

Histamine exerts its biological influence by binding to four specific receptor types, categorized according to their pharmacological responses to various agonists and antagonists. In the context of allergic pathology, the H1 receptor is the primary driver of clinical symptoms. Activation of this receptor leads to several physiological changes, including the contraction of smooth muscles, heightened vascular permeability, and the sensation of itching. It also facilitates prostaglandin production, accelerates atrioventricular node conduction—often manifesting as tachycardia—and stimulates vagal reflexes alongside an increase in cyclic guanosine monophosphate.

The H2 receptor, conversely, regulates different processes such as the secretion of gastric acid and an increase in mucus production within the lower airways.

Symptoms like vasodilation-induced effects and indirect tachycardia resulting from catecholamine release involve a combined activation of both H1 and H2 receptors. The H3 receptor, identified within neurological tissues, is understood to function as a negative feedback regulator for the synthesis and release of histamine [11]. The H4 receptor is predominantly expressed on hematopoietic cells, particularly within immune cell populations—including mast cells, eosinophils, dendritic cells, and T lymphocytes—where it plays a key role in mediating chemotaxis, cytokine release, and inflammatory responses [12]. All the biological effects of histamine are mediated through its interaction with these four receptors, all of which belong to the family of G protein-coupled receptors. H1R is ubiquitously expressed and plays a key role in allergies and inflammation. Histamine mediates immediate hypersensitivity reactions by activating H1R and Gαq/11 [13], which subsequently initiate the activation of phospholipase C β (PLCβ). This enzyme catalyzes the hydrolysis of the membrane phospholipid phosphatidylinositol 4,5-bisphosphate (PIP_2_), resulting in the production of two important second messengers: inositol trisphosphate (IP_3_) and diacylglycerol (DAG). IP_3_ then binds to its receptors located in the endoplasmic reticulum, prompting the release of calcium ions into the cytoplasm. The subsequent elevation of intracellular calcium concentration triggers a series of cellular events mediated by calcium-binding proteins such as calmodulin (CaM), calcineurin, and calcium/calmodulin-dependent kinases [14]. This transient influx activates CaM and myosin light chain kinase (MLCK), driving MLC phosphorylation to facilitate actomyosin complex formation [15,16]. In addition, histamine regulates conjunctival goblet cell mucous secretion through a calcium-dependent signaling pathway [17] and the rearrangement of the cytoskeleton and intercellular gap formation [18].

This pathway highlights the essential role of H1R in orchestrating the immune system’s response to allergens and inflammation [14]. By translating molecular signaling into a broad physiological output, this receptor acts as a key coordinator, mediating all the typical symptoms of the systemic allergic response [19]. Upon binding to the H1 receptor (H1R), histamine mediates respiratory smooth muscle contraction, promotes endothelial hyperpermeability, and stimulates the synthesis of prostacyclin and platelet-activating factor. Therefore, the primary clinical manifestations of immediate hypersensitivity—including cutaneous responses such as erythema, pruritus, and edema—are predominantly driven by H1R activation [13].

This signaling pathway is important because an upregulation of H1R gene expression has been observed in patients with allergic rhinitis. Recent findings indicate that its expression level influences the severity of symptoms and that compounds targeting the H1R gene expression pathway could be useful for developing new antiallergic approaches [20].

Conventional management of allergic conditions primarily relies on antihistamines, intranasal corticosteroids, decongestants, and leukotriene receptor antagonists; nevertheless, the therapeutic landscape is increasingly transitioning toward targeted biological agents directed against IgE and specific cytokines, alongside novel anti-inflammatory modalities and advanced immunotherapeutic [21]. Standard therapeutic regimens exhibit substantial clinical efficacy in managing allergic diseases, but their utility is frequently constrained by specific side effects. Three generations of antihistamines have been developed, characterized by progressively increasing selectivity for the H1 receptor, but in first-generation agents the therapeutic efficacy is significantly constrained by side effects such as sedation, somnolence, and cognitive impairment [22]. Intranasal corticosteroids represent the most effective agents alongside antihistamines [23]; however, their use can lead to local irritation, dryness, and epistaxis [24]. Similarly, leukotriene receptor antagonists are effective in treating rhinitis [25], yet they have been associated with psychiatric manifestations, such as hyperactivity, and abdominal pain [26]. Emerging biological treatments targeting IgE and interleukins show promising immunomodulatory effects [27] and are considered generally well-tolerated [28], though their clinical efficacy varies and some remain in clinical trials [29]. Finally, allergen immunotherapy represents the only disease-modifying option [30].

In addition to standard pharmacological therapies, nutraceutical integration has increasingly emerged as a valuable complementary approach. This integration is particularly relevant given that allergic disorders frequently lead to a significant deterioration in patients’ health-related QoL, stemming both from the persistent clinical manifestations [31] and the adverse effects associated with conventional pharmacological treatments. Within this context, food supplements should complement, not replace, standard medical treatments prescribed by healthcare providers [32]. Due to the numerous side effects associated with the use of medications today, there is a frequent clinical reliance on non-pharmacological interventions aimed at facilitating or expediting recovery from infections and mitigating the exacerbation of allergic symptoms. A diverse range of non-pharmacological products, including dietary supplements, has been established for this purpose. Specific compounds—such as polyphenols—have exhibited potential supportive functions in infection management and immune system modulation. Furthermore, other agents, including quercetin, omega-3 fatty acids, probiotics, and vitamin D, may contribute to the maintenance of respiratory health and the alleviation of symptoms related to allergic respiratory conditions [32]. Nutraceuticals derived from natural sources can effectively modulate immune responses and reduce inflammation in allergic conditions, specifically by downregulating mast cell degranulation [33]. Due to their antioxidant and anti-inflammatory properties, these supplementary foods serve as valuable tools in allergy management [34].

The development of a natural formulation, which comprises Quercetin, *Boswellia serrata*, *Perilla frutescens*, *Helichrysum italicum*, *Ribes nigrum*, and a probiotic complex (*Lactobacillus acidophilus* and *Bifidobacterium animalis*), is guided by available scientific literature suggesting their potential anti-allergic and anti-inflammatory properties in the management of allergic conditions.

Numerous in vitro and in vivo studies have evaluated the therapeutic potential of quercetin in allergic rhinitis (AR). Since elevated serum IgE levels represent a major hallmark of allergic pathology, modulating this humoral response is critical. In vivo evidence from patients suffering from moderate-to-severe AR demonstrated that the administration of a quercetin-containing mixture significantly reduced serum IgE levels [35].

Complementing this effect, rutin, a natural flavonoid highly abundant in *Ribes nigrum*, serves as a potent stabilizer of the innate immune response. Rutin has been shown to inhibit antigen-stimulated mast cell degranulation, while simultaneously downregulating the gene expression and synthesis of key pro-inflammatory cytokines, thereby mitigating mast cell-mediated allergic inflammation [36]. The inclusion of *Helichrysum italicum* within the antiallergic mixture is biologically justified by its dual inhibitory profile on lipid mediator biosynthesis and pro-inflammatory signaling [37]. Furthermore, the inclusion of *Perilla frutescens* provides a targeted approach against IgE-driven type I hypersensitivity. Its bioactive phytocomplex, characterized by highly polyphenolic fractions, effectively suppresses mast cell degranulation by inhibiting Akt phosphorylation and blocking intracellular Ca^2+^ influx, resulting in a robust, dose-dependent inhibition of histamine release [38]. Compared to standard antihistamine therapy, a phytotherapeutic formulation containing *Boswellia serrata* showed superior efficacy in enhancing clinical recovery, decreasing recurrent infections, and mitigating the reliance on corticosteroid and antibiotic therapies in seasonal allergic rhinitis patients [39].

Probiotics mitigate allergic rhinitis (AR) pathophysiology by activating dendritic cells and modulating cytokine networks, strategically downregulating pro-allergic IgE while promoting protective Immunoglobulin G (IgG) antibodies [40]. Translating these immunomodulatory mechanisms into clinical practice, a recent randomized, double-blind, placebo-controlled trial demonstrated that an 8-week intervention with *Bifidobacterium animalis* safely and significantly alleviated perennial AR symptoms. This improvement correlated with a marked suppression of serum total IgE and interleukin-13 (IL-13) levels, confirming the *B. animalis* strain’s therapeutic efficacy in resolving Th2-driven inflammation in allergies without adverse effects [41]. Furthermore, the administration of a multi-strain probiotic consortium comprising *L. acidophilus* has been shown to significantly alleviate clinical symptoms and enhance the overall quality of life in allergic rhinitis patients [42].

It is hypothesized that the synergy of these ingredients may offer an innovative opportunity to modulate the H1 receptor.

A previous investigation, encompassing both in vitro and in vivo models, examined the antihistaminic and anti-inflammatory properties of a nutraceutical mixture composed of Quercetin, *Perilla frutescens*, *Boswellia serrata*, *Ribes nigrum* (Blackcurrant), *Parthenium*, *Helichrysum*, *L. acidophilus*, and *B. animalis*. In the in vitro phase, the formulation demonstrated a statistically significant reduction (Student’s *t*-test) in mast cell (rat leukemia cell line RBL-2H3) degranulation of 35.1%, alongside a 13% decrease in TNF-α levels, suggesting moderate cromon-like anti-inflammatory activity. Furthermore, in vivo evaluations on human subjects revealed antihistaminic effects, characterized by an approximate 30% reduction in wheal diameter [43].

Given that previous research did not fully clarify whether the observed reduction in wheal formation was exclusively attributable to the inhibition of mast cell degranulation, and since the literature demonstrates that quercetin exerts a direct down-regulatory effect on the H1 receptor [44], this study aims to further elucidate the molecular pathways underlying H1 receptor regulation and their specific role in allergic signaling. Specifically, utilizing the same nutraceutical mixture, we seek to determine whether this formulation modulates the H1 receptor, a primary mediator of histamine-induced allergic responses.

## 2. Materials and Methods

The following experiments were performed by NutraTech (Rende, Italy) a Complife group Spin-Off.

### 2.1. Reduction in *H1* Receptor Gene Expression

#### 2.1.1. Experimental Model

The experimental model consisted of human keratinocyte cell cultures (HaCaT) (CLS Cell Lines Service, Cat. No. 300493, Eppellheim, Germany) maintained in Dulbecco’s Modified Eagle Medium (DMEM) supplemented with 10% fetal bovine serum (FBS), 1% L-glutamine, 1% sodium pyruvate, and 1% penicillin/streptomycin. Cells were cultured in a humidified incubator at 37 °C with 5% CO_2_ and 95% relative humidity.

The experiment was conducted in biological triplicate.

#### 2.1.2. Mixture Provenience

The experimental cell model was treated with a specific nutraceutical mixture. Each component of the mixture was obtained from commercial suppliers as follows:

*Boswellia serrata* extract (Akbamax^®^, product code: BSAK-170; 100 mg) was purchased from Arjuna Natural Extracts Ltd. (Alwaye, India); Blackcurrant extract (standardized to 4% rutin; product code: LiP00329; 100 mg) was purchased from Nutraceutica S.r.l. (Ozzano dell’Emilia, Italy); Granular Quercetin (95% purity; product code: FP2254; 210 mg) was purchased from Farezis Pharma S.r.l. (Milano, Italy); Everlasting extract (*Helichrysum* 1/4 flowering tops dry extract; product code: LiP00029; 50 mg) was purchased from Nutraceutica S.r.l. (Ozzano dell’Emilia, Italy); *Perilla frutescens* seed dry extract (standardized to 2.5% polyphenols; product code: obtained as a commercial-grade extract without a specific catalog number; 200 mg) was purchased from Nutratrade S.r.l. (Milano, Italy); Feverfew (*Tanacetum parthenium*) flowering tops dry extract (standardized to 0.5% parthenolide; product code: LiP00306; 100 mg) was purchased from Nutraceutica S.r.l. (Ozzano dell’Emilia, Italy); *Lactobacillus acidophilus* (strain PBS066/LA001-DSM 24936/LMG P-29512; SynBalance^®^ LA 150B, 150 bln/g; 13.3 mg) was purchased from SynBalance S.r.l. (Origgio, Italy); and *Bifidobacterium animalis ssp. lactis* (strain Bi1/deposit number LMG P-17502; 10 mg, equivalent to 1 bln CFU) was purchased from Centro Sperimentale del Latte S.r.l. (Zelo Buon Persico, Italy). For the final concentrations of these ingredients used in the experiments, see Supplementary Materials Table S2.

#### 2.1.3. Treatment and Controls

A total of 1 × 10^6^ cells were seeded in 100 mm culture dishes. After 24 h of incubation, cells were treated with the mixture. Preliminary cytotoxicity assays were performed, identifying 78 μg/mL (see Supplementary Materials Table S1 and Figure S1) as the selected test concentration, as it did not induce cytotoxicity. The mixture was solubilized in Dulbecco’s Modified Eagle Medium (DMEM) supplemented with 10% fetal bovine serum (FBS), 1% L-glutamine, 1% sodium pyruvate, and 1% penicillin/streptomycin.

The experiment included a negative control consisting of cells not treated with any treatment, representing the basal state of the cells.

#### 2.1.4. RNA Extraction

After 24 h of treatment, RNA extraction was performed according to the instructions provided by the utilized kit, PureLink™ RNA Mini Kit (Invitrogen™, Carlsbad, CA, USA). The quantification and quality assessment of RNA were performed using a Take3 Micro Volume Plate and an Epoch microplate reader (BioTek, Winooski, VT, USA) by measuring absorbance ratios at 260/280 nm. Only RNA samples with A260/A280 ratios between 1.8 and 2.0 were considered suitable for qPCR analysis.

#### 2.1.5. Retrotranscription from RNA to cDNA

RNA was quantified, and 2 μg were reverse-transcribed using the High-Capacity cDNA Reverse Transcription Kit (Applied Biosystems™, Waltham, MA, USA). The PCR thermal cycler utilized was Biometra^®^ Tpersonal (Biometra GmbH, Göttingen, Germany).

#### 2.1.6. Quantitative PCR (qPCR)

Gene expression analysis of *HRH1* was performed on the obtained cDNA. Data normalization was carried out using two housekeeping genes, *18S* and *ACTB*.

Primers were provided by Invitrogen and designed as follows:

- *HRH1* Forward (3): CTCTCGAACGGACTCAGATA;
- *HRH1* Reverse: GGAGCCTCTTCCAAGTAAAC;
- *18S* Forward: CGGCGACGACCCATTCGAAC;
- *18S* Reverse: GAATCGAACCCTGATTCCCCGTC;
- *ACTB* Forward: ACAGAGCCTCGCCTTTGCC;
- *ACTB* Reverse: GATATCATCATCCATGGTGAGCTGG.

Relative gene expression was calculated using the 2^−ΔΔCt^ method. Primers were designed using the NCBI Primer-BLAST tool (Primer 3, version 2.5.0) based on *Homo sapiens* mRNA reference sequences. Selection criteria included amplicon lengths ≤ 200 bp, primer pairs spanning exon–exon junctions to avoid genomic DNA amplification, GC content ≥ 40%, and comparable melting temperatures (Tm). Primer specificity was experimentally validated by melting curve analysis, which consistently showed a single specific peak for each target across all samples. For *HRH1*, three primer sets were initially tested, and the best-performing set was selected based on reproducibility and consistent Tm values. Previously validated primer sets were used for the housekeeping genes (*18S* and *ACTB*). The reaction mix was prepared using cDNA and gene-specific primers with PowerUp™ SYBR™ Green Master Mix for qPCR (Applied Biosystems™, Waltham, MA, USA). A total volume of 25 μL of reaction mixture was dispensed into each well of a 96-well PCR plate (0.2 mL semi-skirted, 96-well PCR plate). qPCR analysis was performed using a QuantStudio™ 3 Real-Time PCR System (Applied Biosystems™, Waltham, MA, USA). Specificity of the amplified fragments was confirmed by melting curve analysis, which showed a single peak without evidence of nonspecific amplification or primer-dimer formation. In the no-template controls (NTCs), no amplification was detected, as indicated by undetermined Ct values. No target-specific melting peaks were observed in the NTC wells, supporting the absence of contamination or nonspecific amplification. PCR efficiency was not experimentally determined by standard curve analysis. Relative gene expression was calculated using the 2^−ΔΔCt^ method and statistical comparisons were performed using an unpaired *t*-test with Welch’s correction (GraphPad Prism 10). Differences were considered significant when *p* < 0.05 (* significant vs. Negative Control).

### 2.2. Receptor Activation (*IP3* Dosage)

#### 2.2.1. Experimental Model

The experimental model consisted of human keratinocyte cell cultures (HaCaT) (CLS Cell Lines Service, Cat. No. 300493, Eppellheim, Germany) maintained in Dulbecco’s Modified Eagle Medium (DMEM) supplemented with 10% fetal bovine serum (FBS), 1% L-glutamine, 1% sodium pyruvate, and 1% penicillin/streptomycin. Cells were cultured in a humidified incubator at 37 °C with 5% CO_2_ and 95% relative humidity.

The experiment was conducted in triplicate.

#### 2.2.2. Mixture Provenience

The experimental cell model was treated with a specific nutraceutical mixture. Each component of the mixture was obtained from commercial suppliers as follows:

*Boswellia serrata* extract (Akbamax^®^, product code: BSAK-170; 100 mg) was purchased from Arjuna Natural Extracts Ltd. (Alwaye, India); Blackcurrant extract (standardized to 4% rutin; product code: LiP00329; 100 mg) was purchased from Nutraceutica S.r.l. (Ozzano dell’Emilia, Italy); Granular Quercetin (95% purity; product code: FP2254; 210 mg) was purchased from Farezis Pharma S.r.l. (Milano, Italy); Everlasting extract (*Helichrysum* 1/4 flowering tops dry extract; product code: LiP00029; 50 mg) was purchased from Nutraceutica S.r.l. (Ozzano dell’Emilia, Italy); *Perilla frutescens* seed dry extract (standardized to 2.5% polyphenols; product code: obtained as a commercial-grade extract without a specific catalog number; 200 mg) was purchased from Nutratrade S.r.l. (Milano, Italy); Feverfew (*Tanacetum parthenium*) flowering tops dry extract (standardized to 0.5% parthenolide; product code: LiP00306; 100 mg) was purchased from Nutraceutica S.r.l. (Ozzano dell’Emilia, Italy); *Lactobacillus acidophilus* (strain PBS066/LA001-DSM 24936/LMG P-29512; SynBalance^®^ LA 150B, 150 bln/g; 13.3 mg) was purchased from SynBalance S.r.l. (Origgio, Italy); and *Bifidobacterium animalis ssp. lactis* (strain Bi1/deposit number LMG P-17502; 10 mg, equivalent to 1 bln CFU) was purchased from Centro Sperimentale del Latte S.r.l. (Zelo Buon Persico, Italy). For the final concentrations of these ingredients used in the experiments, see Supplementary Materials Table S2.

#### 2.2.3. Treatment and Controls

A total of 1 × 10^6^ cells were seeded in 100 mm culture dishes. After 24 h of incubation, cells were pre-treated with histamine 100 μM and with the treatment mixture. For the tested mixture, preliminary cytotoxicity assays were performed, identifying 78 μg/mL (see Supplementary Materials Table S1 and Figure S1) as the selected test concentration, as it did not induce cytotoxicity.

The assay included the following controls:

- Negative control: cells not treated with any of the test samples, to represent the basal cellular condition.
- Positive control: cells exposed to histamine only at 100 μM concentration.
- Benchmark: cells exposed to histamine and treated with a known H1 receptor blocker. The benchmark SML2929 BILASTINE (Sigma Aldrich, Saint Louis, MO, USA) was used at a concentration of 3 μM.

#### 2.2.4. IP_3_ Dosage (ELISA Method)

After 24 h of treatment, the culture medium was collected and IP_3_ levels were measured using a specific IP_3_ ELISA kit (Inositol Triphosphate ELISA Kit; Wuhan Elabscience Biotechnology Co., Ltd., Wuhan, China). Prior to analysis, the cell culture medium was centrifuged to remove debris.

The wells provided in this kit were pre-coated with Universal IP_3_. During the reaction, Universal IP_3_ present in the samples or standards competes with a fixed amount of Universal IP_3_ immobilized on the solid-phase support for binding sites on the biotinylated detection antibody specific for Universal IP_3_. Excess conjugate and unbound sample or standard were removed by washing, after which avidin conjugated to horseradish peroxidase (HRP) was added to each microplate well and incubated. Subsequently, a TMB substrate solution was added to each well. The enzyme–substrate reaction was stopped by the addition of a stop solution, and the color change was measured spectrophotometrically at a wavelength of 450 ± 2 nm. The concentration of Universal IP_3_ in the samples was then determined by comparing the optical density (OD) of the samples with the standard curve.

#### 2.2.5. Statistical Analysis of IP_3_ Level

Statistical analysis of IP_3_ levels was performed using ordinary one-way ANOVA with Bonferroni’s multiple comparisons post hoc test (GraphPad Prism 10). Differences were considered statistically significant at *p* < 0.05 (* significant vs. negative control; ° significant vs. Histamine).

## 3. Results

### 3.1. Reduction in *H1* Receptor Gene Expression in *HaCaT* Cell Line

To evaluate the molecular effects of the tested formulation, human HaCaT keratinocytes were treated with the mixture for 24 h. The results obtained from the qPCR are expressed as fold change mean ± standard deviation and are reported in Table 1. Data are normalized to the two housekeeping genes *18S* and *ACTB*.

**Figure 1:**
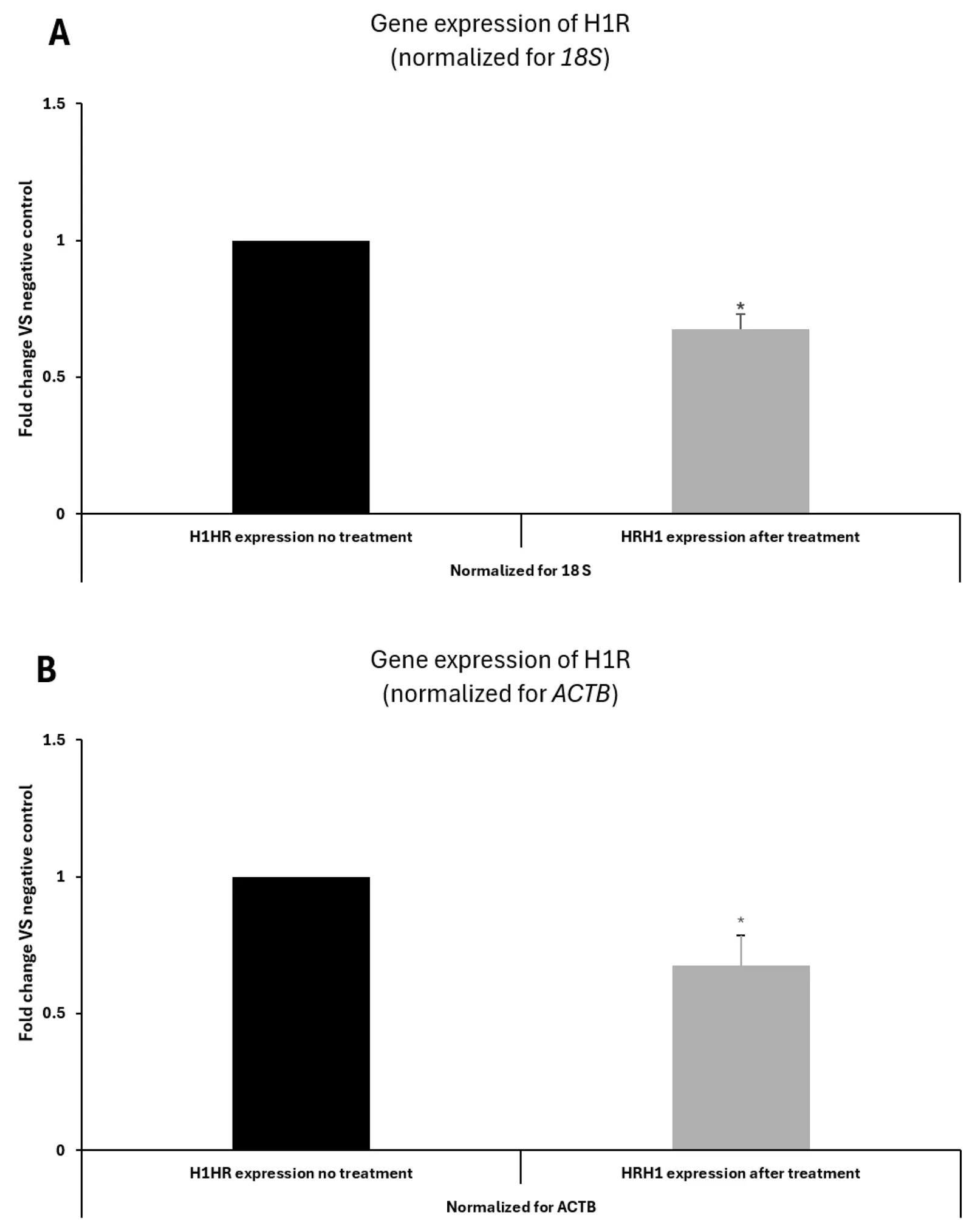
Relative mRNA expression levels of *HRH1* in HaCaT cells. qPCR analysis showing the fold change in *HRH1* expression after 24 h of exposure, normalized against the housekeeping genes (**A**) *18S* and (**B**) *ACTB*. Data are presented relative to the untreated control (set to 1). Relative gene expression was calculated using the 2^−ΔΔCt^ method and statistical comparisons were performed using an unpaired *t*-test with Welch’s correction (GraphPad Prism 10). Differences were considered significant when *p* < 0.05 (* significant vs. Negative Control). (**A**) Significant vs. Negative Control (*p*-value: 0.0104). (**B**) Significant vs. Negative Control (*p*-value: 0.0361).

As can be seen in Figure 1, the results demonstrate that treatment with the tested mixture reduces the expression of the H1 receptor in HaCaT cells by approximately 33% compared with the basal controls, in a statistically significant manner (* *p* < 0.05 vs. negative control, unpaired *t*-test with Welch’s correction on GraphPad Prism 10). The percentage reduction in *HRH1* following treatment with the mixture was remarkably consistent across both housekeeping genes; specifically, a decrease of 32.2% was observed relative to the *18S* control, while a 32.5% reduction was recorded compared to the *ACTB* control.

### 3.2. Inositol Trisphosphate (*IP_3_*) Quantification in *HaCat* Cell Line

**Figure 2:**
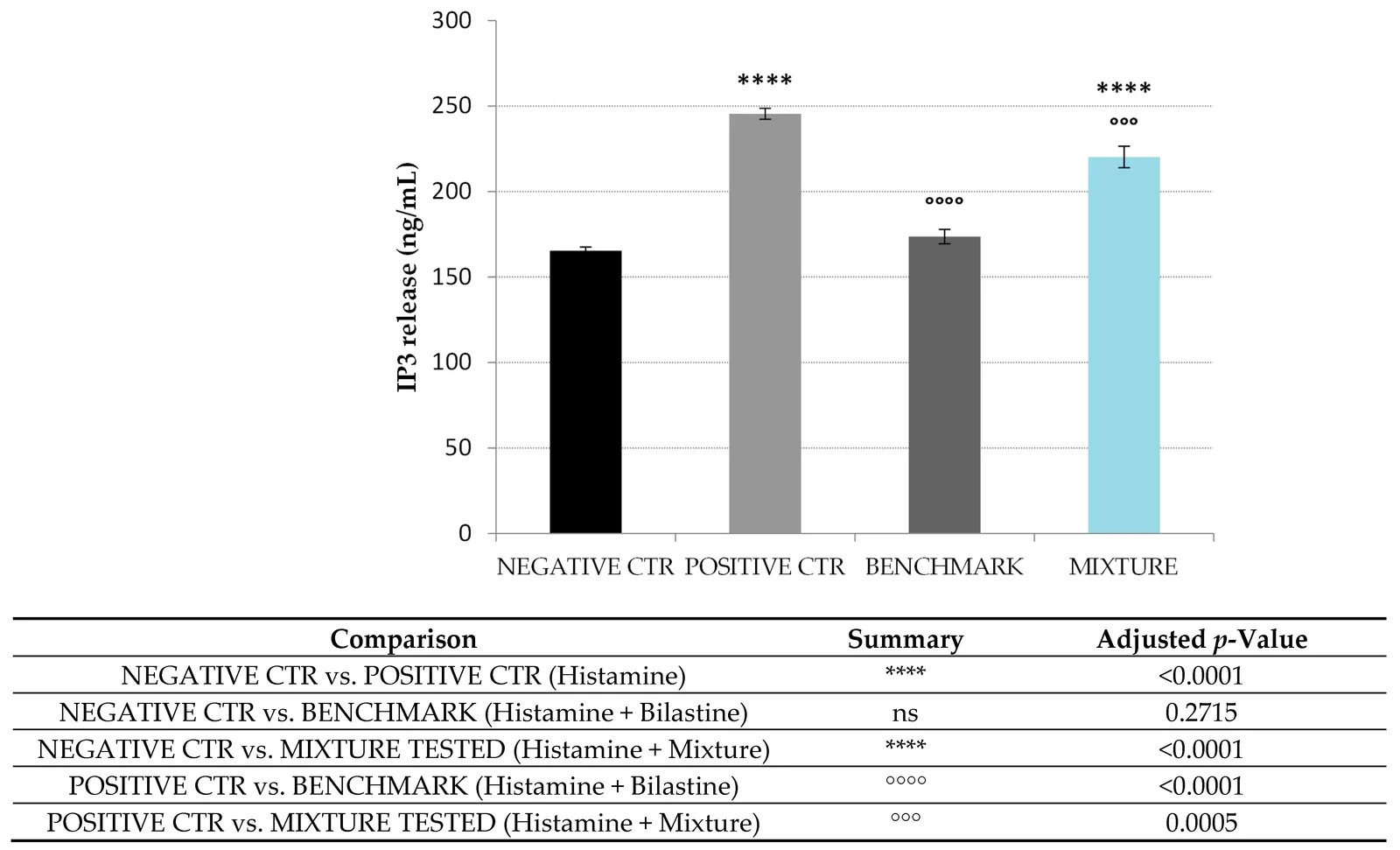
Graphical representation of IP3 release quantification in HaCaT cells. The histogram illustrates the relative changes in IP3 levels under various experimental settings. Bars denote the mean ± SD (*n* = 3). Label. NEGATIVE CTR: untreated cells; POSITIVE CTR: only histamine treatment; BENCHMARK: cells exposed to histamine and treated with the benchmark (bilastine); MIXTURE: cells exposed to histamine and treated with the mixture tested. **** *p* < 0.0001 vs. Negative CTR; °°°° *p* < 0.0001, °°° *p* < 0.001 vs. Positive CTR (ANOVA Bonferroni’s post hoc test). ns, not significant.

To evaluate downstream signaling pathways associated with H1 receptor activation, human HaCaT cells were stimulated with histamine following treatment with either the benchmark or the tested mixture. The results obtained from IP_3_ quantification by ELISA assay are expressed as mean ± standard deviation (SD) and are reported in Table 2. Differences were considered statistically significant at *p* < 0.05 (* significant vs. negative control; ° significant vs. Histamine). The results obtained, shown in Figure 2, demonstrate that histamine treatment significantly increases the levels of IP_3_ released by HaCaT cells compared to the negative control. Treatment with the benchmark significantly reduced IP_3_ release by up to 48.42% with respect to histamine-treated cells, indicating effective receptor blockade. Treatment with the tested mixture resulted in a smaller reduction in IP3 release (up to 10.30%), which was nonetheless statistically significant compared with the positive control of histamine-treated cells.

## 4. Discussion

Allergic diseases represent a growing public health challenge, with conventional pharmacological therapies, although effective, associated with significant side effects.

This study preliminarily elucidates the potential mechanism of action of a nutraceutical mixture—comprising Quercetin, *Perilla frutescens*, *Boswellia serrata*, *Blackcurrant*, *Parthenium*, *Helichrysum*, *Lactobacillus acidophilus*, and *Bifidobacterium animalis*—on H1R gene expression and its pathway.

Compounds that suppress H1R gene expression have shown promise as effective antiallergic drugs. Quercetin possesses numerous biological activities, including antiallergic effects: a study by Hattori et al. (2013) demonstrated that it suppresses the upregulation of H1R gene expression induced by histamine and PMA [44].

Investigations of this mixture previously focused on evaluating the antihistaminic and anti-inflammatory effects of this innovative formulation. The same formulation evaluated in the present study demonstrated a 35.1% in vitro reduction in mast cell degranulation and a 13% decrease in TNF-α levels in RBL-2H3 cell cultures, as well as interference with histamine-induced wheal formation in vivo in human subjects (30%) [43].

While our preliminary data pointed toward a cromone-like stabilization of mast cells, the concurrent reduction in the histamine wheal suggested a secondary antihistaminic-like pathway. This prompted a detailed investigation to elucidate the mixture’s potential mechanism of action, with a specific focus on the modulation of the H1-PLCβ-IP3 signaling pathway.

We conducted experiments using human keratinocyte (HaCaT) cell cultures, a validated model of cutaneous/mucosal barrier function. In fact, several studies have demonstrated the expression and functionality of H1R in human keratinocytes, including HaCaT cells, where histamine activates classical H1R-dependent signaling pathways, including phosphoinositide turnover, intracellular calcium mobilization and downstream inflammatory responses [45,46].

Treatment with the nutraceutical mixture reduced H1 receptor (H1R) gene expression by 32% in HaCaT cells compared to the basal control, with normalization against two housekeeping genes (*18S* and *ACTB*). This finding is noteworthy, as it suggests the mixture may modulate the gene expression of the histamine receptor, thereby influencing the response of the target cells.

In the second part of this study, we quantified inositol trisphosphate (IP_3_) levels. IP_3_ signaling, alongside the diacylglycerol (DAG)–protein kinase C (PKC) pathway, is fundamental to cellular physiology. Upon activation of upstream receptors, PIP_2_ is hydrolysed into IP_3_ and DAG, two key second messengers that regulate diverse cellular processes. DAG activates PKC, leading to protein phosphorylation and varied responses, while IP_3_ induces Ca^2+^ release from non-mitochondrial intracellular stores, exerting multifaceted roles despite its simple structure [47]. Notably, IP_3_ plays a crucial role in the development of allergic symptomatology [19]. For this reason, we investigated how our mixture modulates IP_3_ release relative to differences in cell-derived IP3 levels measured at the end of the treatment period.

In vitro experiments demonstrated that the mixture tested induced a statistically significant 10.30% reduction in IP_3_ levels in histamine-pretreated HaCaT cells compared to the positive control (only treated with histamine).

The possible relationship between IP3 signaling and receptor activation is supported by the fact that histamine treatment, an H1R agonist, triggers an increase in IP3 levels, whereas bilastine (our benchmark in this study)—consistent with its established antagonistic profile—reduces IP3 levels. Consequently, the tested mixture exerts a dual effect: it downregulates H1R expression and probably modulates receptor activity, as evidenced by the significant decrease in IP3 levels.

The inclusion of bilastine as a positive control was useful to demonstrate that it was possible to evaluate an inhibitory effect on the receptor, since bilastine is a notable antagonist. The data obtained indicate that the mixture exhibits a measurable inhibitory effect under the test conditions; however, its activity cannot be considered equivalent to that of bilastine or other conventional drugs. The observed modulation of *HRH1* expression and the differences in cell-derived IP3 levels measured at the end of the treatment suggest that this mixture warrants further preclinical investigation using mast cells as an experimental model, followed by clinical studies in patients with allergic diseases. In conclusion, this work highlights the importance of preclinical research targeting the H1 receptor for the development of innovative natural products aimed at managing allergic disorders.

## 5. Conclusions

The in vitro study suggests that the innovative nutraceutical mixture—comprising Quercetin, *Perilla frutescens*, *Boswellia serrata*, Blackcurrant, *Parthenium*, *Helichrysum*, *Lactobacillus acidophilus*, and *Bifidobacterium animalis*—may modulate the histamine H1 receptor pathway in HaCaT cells. Treatment significantly downregulated *HRH1* gene expression and reduced IP3 release in histamine-treated cells, suggesting an interference with the H1R-PLCβ-IP3 signaling.

When integrated with prior research on this specific formulation, the present results contribute to a broader understanding of its pharmacological profile in vitro. Nevertheless, the translation of these findings to in vivo systems warrants further exploration. Future research should prioritize randomized clinical trials to evaluate symptomatic efficacy, bioavailability, and long-term immunomodulatory effects in allergic patients.

## Figures and Tables

**Table 1:** Gene expression analysis by qPCR on HaCaT cells. Data represent the relative fold change compared to the control group (normalized to *18S* and *ACTB* genes). Values are expressed as mean ± SD (*n* = 3). Differences were considered significant when *p* < 0.05 (* significant vs. Negative Control).

	Normalized for *18S*		Normalized for *ACTB*	
	NEGATIVE CTR	*HRH1*	NEGATIVE CTR	*HRH1*
FOLD CHANGE	1	0.674	1	0.675
DEV. ST		0.058164		0.109683
%VAR vs. CTR-		−32.2% *		−32.5% *
*p*-value		0.0104		0.0361

**Table 2:** Quantitative analysis of IP3 release in human HaCaT cells. Data represent the absolute or relative quantification of IP3 release across different experimental conditions. Values are expressed as mean ± standard deviation (SD) (*n* = 3). Statistical analysis of IP3 levels was performed using ordinary one-way ANOVA with Bonferroni’s multiple comparisons post hoc test (GraphPad Prism 10). Differences were considered statistically significant at *p* < 0.05 (**** significant vs. negative control; °°°°/°°° significant vs. Histamine;).

	IP_3_ Release Mean Value (ng/mL)	Dev. St	%VAR VS CTR−	%VAR VS CTR+
NEGATIVE CTR	165.41	2.14		
POSITIVE CTR	245.5	3.15	48.42% ****	
BENCHMARK	173.65	4.23	4.98% ns	−29.26% °°°°
MIXTURE TESTED	220.22	6.3	33.13% ****	−10.3% °°°

## Data Availability

The original contributions presented in this study are included in the article and in the Supplementary Material File. Further inquiries can be directed to the corresponding author.

## Supplementary Materials

The following supporting information can be downloaded at https://www.mdpi.com/article/10.3390/cimb48090854/s1.
