# Supplementary material for: Preclinical In Vitro Evaluation of an Innovative Nutraceutical Mixture Modulating the Histamine H1 Receptor—Preliminary Data
Source: Curr Issues Mol Biol. 2026 Aug 22;48(9):854. doi: 10.3390/cimb48090854 (PMC13605393; doi:10.3390/cimb48090854)
Supplement: Supplementary file 1 [file cimb-48-00854-s001.zip › cimb-4442868-supplementary.pdf]

current issues in molecular biology

MDPI

Supplementary Materials

Preclinical In Vitro Evaluation of an Innovative Nutraceutical Mixture Modulating the Histamine H1 Receptor—Preliminary Data

Franco Frati [1], Carlo Cavaliere [2], Francesco Frati [3], Giulio Torello [4], Marianna Colasante [4], Chiara Caputo [5], Marta Scquizzato [5,*] and Simonetta Masieri [6]

[1] Private Independent Researcher, 20123 Milano, Italy; francofrati57@gmail.com
[2] Department of Sense Organs, Sapienza University, 00161 Rome, Italy; carlo.cavaliere@uniroma1.it
[3] Clinic of Otolaryngology (ENT), University of Perugia, 06123 Perugia, Italy; francescofrati92@hotmail.it
[4] TL Pharma Consulting, 65129 Pescara, Italy; giulio.torello@tlpharmaconsulting.it (G.T.); ricerca@tlpharmaconsulting.it (M.C.)
[5] Science Department Global Pharmacies Partner, 20123 Milano, Italy; c.caputo@gppitaly.it
[6] Department of Oral and Maxillofacial Sciences, Sapienza University, 00161 Rome, Italy; studiomasieri@gmail.com
* Correspondence: m.scquizzato@gppitaly.it

In the absence of available pharmacokinetic data, we hypothesized a model assuming 100% bioavailability and distribution restricted exclusively to the blood compartment (without accounting for hepatic passage or tissue biodistribution). Experimental concentration was determined based on the calculation of the theoretical maximal blood concentration, assuming a blood volume of 5 L in an average adult subject. Considering a mixture weight of approximately 780 mg and assuming complete distribution within the blood compartment, the theoretical blood concentration was estimated at 156 µg/mL. Based on this value, preliminary cytotoxicity assays were performed, identifying 78 µg/mL as the selected test concentration, as it did not induce cytotoxic. The concentrations were prepared in Dulbecco's Modified Eagle Medium (DMEM) immediately before treatment.

The HaCaT keratinocyte cell line (CLS Cell Lines Service, Cat. No. 300493) was employed to evaluate cytotoxicity via the MTT [3-(4,5-dimethylthiazol-2-yl)-2,5-diphenyltetrazolium] assay. HaCaT cells were seeded in 96-well plates at a density of 1.5 × 10^4 cells/well in complete medium and incubated for 24 h.

The mixture was pulverized using a sterile mortar and pestle to obtain a homogeneous powder. To ensure the complete dissolution of the hydrophobic botanical compounds (e.g., Quercetin and Boswellia serrata extract), the powder was initially suspended in Dimethyl sulfoxide (DMSO). The primary solution was subsequently diluted in complete Dulbecco's Modified Eagle Medium (DMEM) to reach the stock concentration of 156 µg/mL. To facilitate optimal homogenization, the mixture was sonicated at room temperature. The stock solution (156 µg/mL) was prepared in Dulbecco's Modified Eagle Medium (DMEM) supplemented with 10% FBS, 1% L-glutamine, 1% sodium pyruvate, and 1% penicillin/streptomycin. This initial concentration was determined based on a theoretical maximum blood concentration, assuming an average adult blood volume of 5 L and the total systemic absorption of a 780 mg dose. Eight serial dilutions (1:2) were subsequently prepared.

After 24 h of treatment, 200 µL of MTT solution (0.3 mg/mL) was added to each well and incubated for 3 h at 37°C. The supernatant was then replaced with 200 µL of DMSO

 https://doi.org/10.3390/cimb48090854

to dissolve the resulting formazan crystals. Absorbance was recorded at 570 nm using a Beckman Coulter microplate reader. All experiments were performed in triplicate.

Preliminary results, summarized in Table S1 and Figure S1, identified 78 µg/mL as the optimal non-cytotoxic dosage, maintaining a cell viability of 90.34% ± 0.28. The table S2 report the specific final concentrations of each individual component of the mixture in the testing medium at the same dosage.

Table S1. Data obtained from preliminary citotoxicity in terms of cell viability expressed as mean ± standard deviation on HaCat cell line. Experiments were performed in triplicate.

Conc. of the mixture (µg/mL)	Cell Viability %	dev. St
156	74.72	0.48
78	90.34	0.28
39	92.47	0.12
19.5	92.51	0.06
9.75	97.09	0.06
4.875	101.11	0.06
2.4375	109.01	0.15
1.21875	106.46	0.23
Negative control	100	0.2

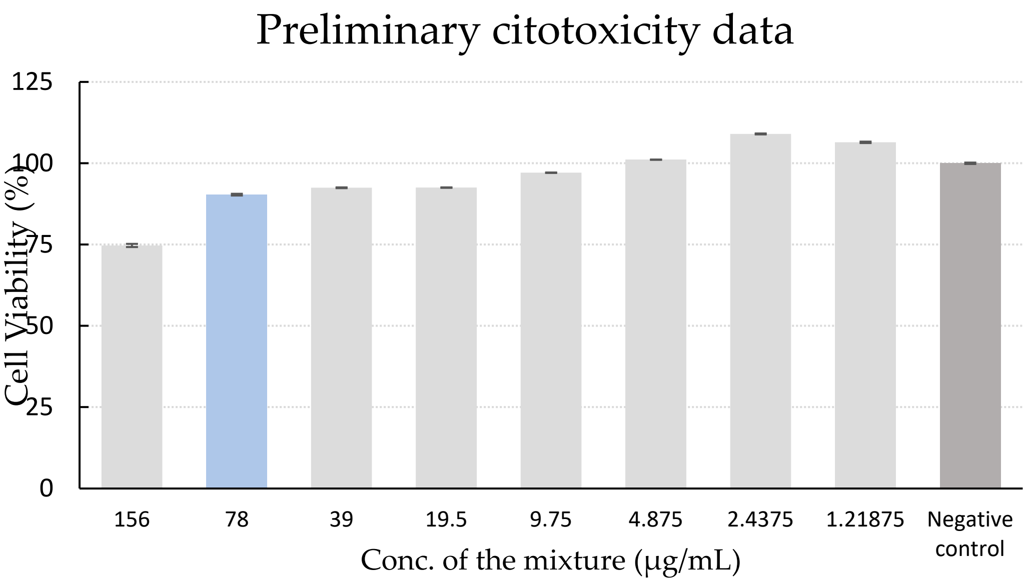

Figure S1. Preliminary cytotoxicity profile of the tested compound on HaCat cell line. The bar graph illustrates the cell viability percentage across a range of concentrations from 1.21875 to 156 µg/mL. The gray bar represents the negative control (100% viability). Error bars indicate the standard deviation.

https://doi.org/10.3390/xxxxx

Table S2. Composition and final concentrations of the tested mixture in the cell culture medium. Values correspond to the optimal non-cytotoxic dosage of 78 μg/mL applied to the HaCaT keratinocyte cell line. (d.e.: dry extract).

Ingredients	Amount (mg)	Proportions in the mixture	Ingredient Concentration at dilution of 78 μg/mL
Quercetin (95% granular)	210	26.81 %	20.91
Perilla leaves d.e. (2.5% polyphenols)	200	25.53 %	19.92
Boswellia serrata (Akbamax) d.e. (75%)	100	12.77 %	9.96
Ribes leaves d.e. (4% rutin)	100	12.77 %	9.96
Partenio d.e. (0.5% parthenolides)	100	12.77 %	9.96
Elicriso d.e. 1/4	50	6.38 %	4.98
L. acidophilus (150 MLD/g)	13.3	1.70 %	1.32
B. animalis subsp. lactis (200 MLD/g)	10	1.28 %	1
